# Chinese Patent Medicine Liu Wei Di Huang Wan Combined with Antihypertensive Drugs, a New Integrative Medicine Therapy, for the Treatment of Essential Hypertension: A Systematic Review of Randomized Controlled Trials

**DOI:** 10.1155/2012/714805

**Published:** 2012-11-11

**Authors:** Jie Wang, Kuiwu Yao, Xiaochen Yang, Wei Liu, Bo Feng, Jizheng Ma, Xinliang Du, Pengqian Wang, Xingjiang Xiong

**Affiliations:** ^1^Department of Cardiology, Guang′anmen Hospital, China Academy of Chinese Medical Sciences, Beijing 100053, China; ^2^Department of Gastroenterology, Guang′anmen Hospital, China Academy of Chinese Medical Sciences, Beijing 100053, China; ^3^Graduate School, China Academy of Chinese Medical Sciences, Beijing 100700, China; ^4^Department of Endocrinology, Traditional Chinese Medicine Hospital of Mentougou District, Beijing 102300, China

## Abstract

*Objectives*. To assess the beneficial and adverse effects of Liu Wei Di Huang Wan (LWDHW), combined with antihypertensive drugs, for essential hypertension. *Methods*. Five major electronic databases were searched up to August 2012 to retrieve any potential randomized controlled trials designed to evaluate the clinical effectiveness of LWDHW combined with antihypertensive drugs for essential hypertension reported in any language, with main outcome measures as blood pressure. The quality of the included studies was assessed with the Jadad scale and a customized standard quality assessment scale. *Results*. 6 randomized trials were included. The methodological quality of the trials was evaluated as generally low. The pooled results showed that LWDHW combined with antihypertensive drugs was more effective in blood pressure and the scale for TCM syndrome and symptom differentiation scores compared with antihypertensive drugs alone. Most of the trials did not report adverse events, and the safety is still uncertain. *Conclusions*. LWDHW combined with antihypertensive drugs appears to be effective in improving blood pressure and symptoms in patients with essential hypertension. However, the evidence remains weak due to the poor methodological quality of the included studies.

## 1. Introduction

Hypertension is one of the most prevalent and important public health concerns in both developed and developing countries [1–3]. Several studies have demonstrated that successful long-term treatment of hypertension has a significant impact on morbidity and mortality for cardiovascular disease (CVD) and stroke. However, only 53% of patients treated for hypertension had blood pressure actually controlled to ≤140/90 mmHg [4]. Considering these data and the seriousness of the effects of hypertension on the individual and society as a whole, both economically and socially, physicians must look for more effective and alternative ways to achieve the target blood pressure as quickly as possible. Can integrative medicine contribute to combat hypertension more effectively?

Integrative medicine is a relative new discipline which attempts to combine complementary and alternative medicine (CAM) with Western medicine [5]. As Western medicine has been developed based on the scientific method, integrative medicine, therefore, combines the latest modern scientific advances with the most profound perspectives of CAM to regain and preserve health [6]. In China, the integrative medicine mainly refers to the integrated traditional Chinese and Western medicine. Traditional Chinese medicine (TCM), including herbal medicine and acupuncture, is one of the most important parts in CAM. TCM has long been used in the treatment of a wide variety of illnesses including hypertension [7, 8]. Much of the past literature reflects clinical observations made by physicians in their offices. More recently, with more and more reports of the significance of disease-syndrome combination [9], there are also large numbers of controlled studies based on the use of combination of TCM and Western medicine in the treatment of hypertension [10–14].

Liu Wei Di Huang Wan (LWDHW), a traditional Chinese patent medicine containing six commonly used herbs (*Rehmanniae radix*, pulp of *Cornus*, yam, *Poria cocos*, *Alisma orientalis*, and *Cortex moutan*), is widely used to treat hypertension-related signs and symptoms in clinical practice for centuries in China. Recent research showed that LWDHW could lower blood pressure. The mechanism of the prescription maybe related to enriching yin and nourishing kidney in TCM. Biochemically, LWDHW also showed good effect in decreasing the concentrations of ET and vWF in plasma, increasing the content of NO and protecting the function of kidney with essential hypertension [15, 16]. 

Our previous studies have showed that kidney deficiency syndrome is one of the most important pathogenesis of essential hypertension in TCM, which could be well treated by LWDHW [17]. LWDHW combined with antihypertensive drugs, a new integrative medicine therapy, has been widely used as an alternative and effective method for treating essential hypertension in China currently. A large number of clinical studies reported the clinical effect of LWDHW and LWDHW combined with antihypertensive drugs ranging from case reports and case series to controlled observational studies and randomized clinical trials (RCTs) until now. However, there is no a critically appraised evidence such as systematic reviews or meta-analyses on potential benefit and safety of LWDHW combined with antihypertensive drugs for essential hypertension to justify their clinical use and their recommendation. Understanding the effect of LWDHW combined with antihypertensive drugs on blood pressure, hypertension-related signs and symptoms, quality of life (QOF), and cardiovascular risk factors could be valuable for essential hypertension management. The present paper aims to evaluate the beneficial and harmful effects of LWDHW combined with antihypertensive drugs for treatment of essential hypertension in randomized trials. 

## 2. Methods

### 2.1. Database and Search Strategies

The literature searches were conducted in Chinese National Knowledge Infrastructure (CNKI), Chinese Scientific Journal Database (VIP), Chinese Biomedical Literature Database (CBM), PubMed, and the Cochrane Central Register of Controlled Trials (CENTRAL) in the Cochrane Library (August, 2012). All of those searches ended on 10 August, 2012. Ongoing registered clinical trials were searched in the website of Chinese clinical trial registry (http://www.chictr.org/) and international clinical trial registry by the US national institutes of health (http://clinicaltrials.gov/). The following search terms were used individually or combined: “essential hypertension”, “hypertension,” “Liu Wei Di Huang Wan,” “Liu Wei Di Huang Pill,” “clinical trial,” and “randomized controlled trial.” The bibliographies of the included studies were searched for additional references. 

### 2.2. Inclusion Criteria

 All the randomized controlled trials (RCTs) of all the prescriptions based on “Liu Wei Di Huang Wan” combined with antihypertensive drugs compared with antihypertensive drugs in patients with hypertension were included. There were no restrictions on population characteristics, language, and publication type. The primary outcome measure was blood pressure (BP), and the secondary outcome measure was the scale for TCM syndrome and symptom differentiation (TCM-SSD) scores. The criteria “significant effective, effective, or not effective” was also included in the outcome measurement. Duplicated publications reporting the same groups of participants were excluded.

### 2.3. Data Extraction and Quality Assessment

Two authors conducted the literature searching (X. J. Xiong, K. W. Yao), study selection (X. J. Xiong, B. Feng), and data extraction (X. J. Xiong, X. Du) independently. The extracted data included authors, title of study, year of publication, study size, age and sex of the participants, details of methodological information, name and component of Chinese herbs, treatment process, details of the control interventions, outcomes (e.g., blood pressure), and adverse effects for each study. Disagreement was resolved by discussion and reached consensus through a third party (J. Wang). The methodological quality of trials was assessed using the 6 criteria 6 election bias (study design, confounders, blinding, data collection methods, withdrawals, and dropouts) to follow 3 categories: Category A (strong quality): four strong ratings with no weak ratings above. Category B (moderate quality): less than four strong ratings and one weak rating. Category C (weak quality): two or more weak ratings. The quality of included trials were assessed according to the *Cochrane Handbook of Systematic Reviews of Interventions* (Chapter 8.5) to address the following five criteria: sequence generation, allocation concealment, blinding, incomplete outcome data, selective outcome reporting, and other sources of bias.

### 2.4. Data Synthesis

Revman 5.1 software provided by Cochrane Collaboration was used for data analyses. Dichotomous data were expressed as relative risk (RR) and continuous outcomes as weighted mean difference (WMD), both with 95% confidence intervals (CI). Meta-analysis was performed if the intervention, control, and outcome were the same or similar. The statistical heterogeneity was presented as significant when *I* square (*I* ^2^) is over 50% or *P* < 0.1. In the absence of significant heterogeneity, we pooled data using a fixed-effect model (*I* ^2^ < 50%), otherwise we used random effects model (*I* ^2^ > 50%) [24].

## 3. Result

### 3.1. Description of the Included Trials

**Figure 1:**
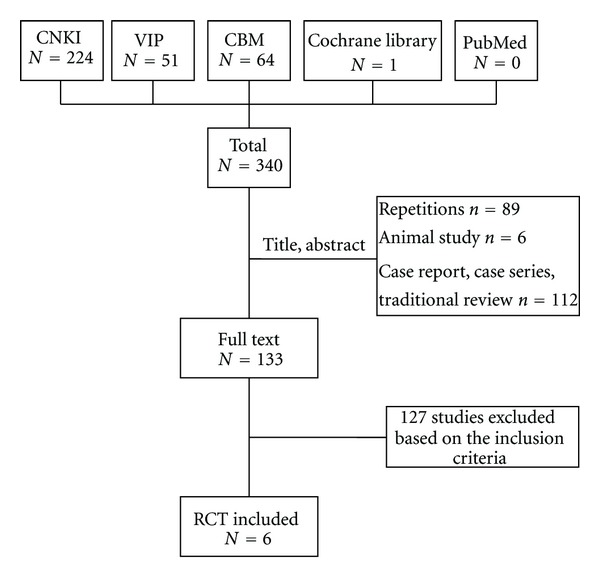
Study selection process.

After primary search of 5 databases, 340 trials were screen out from electronic and manual searches as shown in Figure 1, and the majority were excluded due to obvious ineligibility which included irrelevant titles and abstracts (some papers being found from more than one database). After reading the titles and abstracts, a majority of studies were excluded, and only 133 studies were got. 89 trials were excluded because of duplicated publication, 6 trials were excluded due to the animal studies, and the rest 112 trials were noncontrolled clinical trials including case report, case series traditional review. 127 out of the rest of 133 articles were excluded based on the inclusion criteria. In the end, 6 RCTs were reviewed [18–23]. All the trials were conducted in China and published in Chinese. The characteristics of 6 randomized trials were summarized in Table 1. 

The 6 RCTs involved 555 patients with essential hypertension. There was a wide variation in the age of subjects (33–78 years). 6 trials specified three diagnostic criteria of hypertension, two trials [18, 22] used 1999 WHO-ISH guidelines for the management of hypertension (1999 WHO-ISH GMH), one trial [20] used Chinese Guidelines for the Management of Hypertension-2005 (CGMH-2005), one trial [21] used 1993 WHO-ISH guidelines for the management of hypertension (1993 WHO-ISH GMH), and two trials [19, 23] only demonstrated patients with essential hypertension. Only one trial [23] demonstrated patients with yin-deficiency and excessive yang syndrome in TCM, and the rest trials have not reported TCM diagnostic criteria yet.

The interventions of all the trials [18–23] included LWDHW combined with antihypertensive drugs as shown in Table 1. The controls included antihypertensive drugs alone. The total treatment duration ranged from 4 weeks to 18 weeks. All of the 6 trials used the blood pressure (BP) as the main outcome measure. Other outcome measures include the scale for TCM syndrome and symptom differentiation (TCM-SSD). Adverse effect was described in details. Three classes were used to evaluate treatment effects, including significant effective, effective, ineffective according to BP and TCM-SSD.

### 3.2. Methodological Quality of Included Trials

The methodological quality of all the included RCTs was assessed to be of general low according to the predefined quality assessment criteria as shown in Table 2. The randomized allocation of participants, allocation concealment and double-blind were not mentioned in all the six trials. Only one trial [18] has reported drop-out. None of trials had a pre-trial estimation of sample size, which indicated the lack of statistical power to ensure appropriate estimation of the therapeutic effect. All the trials did not mention follow up. We have tried to contacte the authors for further detailed information about the article, but regrettably no information could be got till now.

### 3.3. Effect of the Interventions

#### 3.3.1. Blood Pressure

Three trials [18, 20, 21] used blood pressure decrease to measure the outcome: significant effective (diastolic blood pressure decreased by 10 mmHg reaching the normal range, or diastolic blood pressure has not yet returned to normal, but has been reduced ≥20 mmHg), effective (diastolic blood pressure decreased to less than 10 mmHg reaching the normal range, or diastolic blood pressure decreased by 10–19 mmHg, but did not reach the normal range, or systolic blood pressure decreased ≥30 mmHg), and ineffective (not to meet the above standards). The trial showed significant difference between treatment and control group on the three criteria outcome measurement (RR: 2.77 (1.37, 5.57); *P* = 0.004). Five trials 19-21, 23-24 compared the effectiveness using the blood pressure value, and significant difference was found between treatment and control group in systolic blood pressure (WMD: −9.31 (−10.75, −7.86); *P* < 0.00001) and diastolic blood pressure (WMD: −6.27 (−7.69, −4.86); *P* < 0.00001) (Tables 3, 4, and 5). 

#### 3.3.2. TCM-SSD Scores

Two trials [21, 22] used the TCM-SSD scores to measure the outcome: significant effective (The main symptoms such as headache, dizziness, palpitations, insomnia, tinnitus, and irritability disappear, or TCM-SSD scores reduced rate ≥70%), effective (the main symptoms relieved, or 70% > TCM-SSD scores reduced rate ≥30%), and ineffective (the main symptoms do not change, or TCM-SSD scores reduced rate <30%). There is only one trial [21] who reported the TCM-SSD scores decrease. The meta-analysis showed there is significant beneficial effect on the combination group compare to the antihypertensive drugs using alone (RR: 3.04 1.10, 8.38; *P* = 0.03) (Table 6). The other trial [22] reported that after 18 weeks of treatment, the scores of main symptoms in hypertension, including headache, insomnia, amnesia, waist soreness, and tinnitus, decreased significantly. As we cannot obtain more details of the TCM-SSD scores, so we cannot get the analysis of comparison between groups (Table 6).

#### 3.3.3. Other Outcomes

One trial [18] showed that after 8 weeks of treatment, the serum level of ET decreased and NO increased in both groups with significant difference in LWDHW plus sustained-release nifedipine group compared to sustained-release nifedipine group. One trial [19] showed that after 4 weeks of treatment, *β*_2_-MG reduced significantly in treatment group compared to control group. One trial [20] showed that significant difference was found between treatment and control group in BUN and Cr after 8 weeks of treatment. One trial [22] reported that significant difference was found between treatment and control group in IgG and C_3_ after 18 weeks of treatment. One trial [23] showed that IgG and C_3_ decreased in the treatment group. 

### 3.4. Sensitivity Analysis, Subgroup Analysis, and Publication Bias

The number of trials was too small to conduct any sufficient additional analysis of sensitivity, subgroup, and publication bias.

### 3.5. Adverse Effect

Only one trial mentioned the adverse effect [20]. It mentioned adverse effect such as dry cough during the course. It may be related to the adverse effect of captopril. 

## 4. Discussion

This paper included 6 randomized trials and a total of 555 participants. Liu Wei Di Huang Wan combined with antihypertensive drugs, a new integrative medicine therapy, showed significant benefit on decreasing blood pressure and improving symptoms and signs as compared with conventional treatment for essential hypertension. However, due to the low-quality methodology and potential publication bias, a definite conclusion of the beneficial effectiveness of LWDHW combined with antihypertensive drugs in treating essential hypertension could not be drawn. The positive findings should be interpreted conservatively due to the following facts. 

All the six trials included had improper study design or method used, such as lack of randomization or allocation concealment. No trials reported randomization procedure clearly and only mentioned that “patients were randomized into two groups”. So, it is hard to judge whether randomization was conducted properly and really. All the trials haven't mentioned allocation concealment and double-blind. Therefore, it is possible that some of the trials are not true RCTs. And two trials including Cai 2004 and Zhang 2007 [18, 19], only have one author. It is impossible for an RCT to be done properly in terms of randomization and allocation concealment by one doctor totally. Publication bias could also be a factor. We have tried to take all measures to contact authors to get further information either by telephone or email. Unfortunately, no replies and informations was got. Subsequently, no clear conclusion could be made from these trials.

Adverse effect was reported by Chen et al. in 2008 [20] as only dry mouth. The adverse effect was not severe, and it spontaneously recovered without special treatment. The other 5 trials did not report any adverse effects and these were not significantly different between the two treatments. Therefore, due to the limited and inadequate evidence provided by the eligible trials, conclusions about the safety of LWDHW combined with antihypertensive drugs cannot be made from this paper. Large-scale clinical trials with long-term followup were warranted to assess the safety of new integrative medicine therapy properly.

Syndrome is a classification according to subjective symptom and objective sign collected by physician through inspection, auscultation-olfaction, interrogation and palpation [25]. Syndrome differentiation is the basic rule in TCM. Failure to apply syndrome differentiation to clinical trials may result in treatments being ineffective or even harmful, and failure to evaluate the real efficacy of TCM. In this paper, only one trial [23] reported the TCM diagnostic criteria but without further details. The rest five trials [18–22] did not report TCM diagnostic criteria. If syndrome differentiation in TCM was considered into the studies properly, the positive effects could be enhanced. 

## 5. Conclusions

In conclusion, because of the unclear methodological quality of these including trials, a definite conclusion on efficacy and safety associated with Liu Wei Di Huang Wan combined with antihypertensive drugs cannot be drawn from this paper. Before recommending Liu Wei Di Huang Wan combined with antihypertensive drugs as an alternative treatment measure in hypertensive patients, more rigorous trials with high quality are needed to give high level of evidence.

## Figures and Tables

**Table 1:** Characteristics and methodological quality of the included studies.

Study ID	Sample	Diagnosis standard	Intervention	Control	Course (week)	Outcome measure
Cai 2004 [18]	162	1999 WHO—ISH GMH	LWDHW plus sustained-release nifedipine	sustained-release nifedipine	8	BP
Zhang 2007 [19]	68	Hypertension diagnostic criteria (unclear)	LWDHW plus nifedipine controlled release tablet	Nifedipine controlled release tablet	4	BP
Chen et al. 2008 [20]	97	CGMH-2005	LWDHW plus captopril	captopril	8	BP; adverse effect
Zhang et al. 2004 [21]	78	1993 WHO—ISH GMH	LWDHW plus enalapril	enalapril	7	BP; TCM-SSD
Zhou et al. 2003 [22]	100	1999 WHO—ISH GMH	LWDHW plus nifedipine controlled release tablet	Nifedipine controlled release tablet	18	BP; TCM-SSD
Hu et al. 1994 [23]	50	Hypertension diagnostic criteria (unclear); TCM diagnostic criteria (unclear)	LWDHW plus nifedipine	nifedipine	10	BP

**Table 2:** Quality assessment of the included randomized controlled trials.

Included trials	Sequence generation	Allocation concealment	Blinding of participants personnel and outcome assessors	Incomplet outcome data	Selective outcome reporting	Other sources of bias	Risk of bias
Cai 2004 [18]	Unclear	Unclear	Unclear	Yes	No	Unclear	High
Zhang 2007 [19]	Unclear	Unclear	Unclear	Yes	No	Unclear	High
Chen et al. 2008 [20]	Unclear	Unclear	Unclear	Yes	No	Unclear	High
Zhang et al. 2004 [21]	Unclear	Unclear	Unclear	Yes	No	Unclear	High
Zhou et al. 2003 [22]	Unclear	Unclear	Unclear	Yes	No	Unclear	High
Hu et al. 1994 [23]	Unclear	Unclear	Unclear	Yes	No	Unclear	High

**Table 3:** Analyses of blood pressure.

Trials		Intervention (*n*/*N*)	Control (*n*/*N*)	RR (95% CI)	*P* value
LWDHW plus sustained-release nifedipine versus sustained-release nifedipine	1	90/93	52/61	5.19 (1.35, 20.04)	0.02
LWDHW plus captopril versus captopril	1	45/49	43/48	1.31 (0.33, 5.20)	0.70
LWDHW plus enalapril versus enalapril	1	35/42	23/36	2.83 (0.98, 8.15)	0.05

Meta-analysis	3	170/184	118/145	2.77 (1.37, 5.57)	0.004

**Table 4:** Analyses of systolic blood pressure.

Trials		WMD (95% CI)	*P *value
LWDHW plus sustained-release nifedipine versus sustained-release nifedipine	1	−10.00 (−11.76, −8.24)	<0.00001
LWDHW plus nifedipine controlled release tablet versus nifedipine controlled release tablet	1	−10.50 (−24.07, 3.07)	0.13
LWDHW plus captopril versus captopril	1	−9.00 (−13.66, −4.34)	0.0002
LWDHW plus nifedipine controlled release tablet versus nifedipine controlled release tablet	1	−8.00 (−11.35, −4.65)	<0.00001
LWDHW plus nifedipine versus nifedipine	1	−3.00 (−10.91, 4.91)	0.46

Meta-analysis	5	−9.31 (−10.75, −7.86)	<0.00001

**Table 5:** Analyses of diastolic blood pressure.

Trials		WMD (95% CI)	*P* value
LWDHW plus sustained-release nifedipine versus sustained release nifedipine	1	−7.00 (−8.74, −5.26)	<0.00001
LWDHW plus nifedipine controlled release tablet versus nifedipine controlled release tablet	1	−2.25 (−11.90, 7.40)	0.65
LWDHW plus captopril versus captopril	1	−6.75 (−11.26, −2.24)	0.003
LWDHW plus nifedipine controlled release tablet nifedipine controlled release tablet	1	−4.00 (−7.23, −0.77)	0.02
LWDHW plus nifedipine versus nifedipine	1	−6.00 (−14.46, 2.46)	0.16

Meta-analysis	5	−6.27 (−7.69, −4.86)	<0.00001

**Table 6:** Analyses of TCM-SSD scores.

Trials		Intervention (*n*/*N*)	Control (*n*/*N*)	RR (95% CI)	*P *value
LWDHW plus enalapril versus enalapril	1	34/42	21/36	3.04 (1.10, 8.38)	0.03
Meta-analysis	1	34/42	21/36	3.04 (1.10, 8.38)	0.03
